# RADEX: a rule-based clinical and radiology data extraction tool demonstrated on thyroid ultrasound reports

**DOI:** 10.1007/s00330-025-11416-4

**Published:** 2025-02-13

**Authors:** Lewis Howell, Amir Zarei, Tze Min Wah, James H. Chandler, Shishir Karthik, Zara Court, Helen Ng, James R. McLaughlan

**Affiliations:** 1 School of Computing, University of Leeds, Leeds, LS2 9JT UK; 2 School of Electronic and Electrical Engineering, University of Leeds, Leeds, LS2 9JT UK; 3 Department of Radiology, Leeds Teaching Hospitals NHS Trust, Leeds, LS9 7TF UK; 4 Leeds Institute of Medical Research, University of Leeds, St James’ University Hospital, LS9 7TF Leeds, UK; 5 Leeds Teaching Hospitals NHS Trust, Leeds, LS9 7TF UK

**Keywords:** Natural Language Processing, Information extraction, Data annotation, Ultrasound, Thyroid

## Abstract

**Objectives:**

Radiology reports contain valuable information for research and audits, but relevant details are often buried within free-text fields. This makes them challenging and time-consuming to extract for secondary analyses, including training artificial intelligence (AI) models.

**Materials and methods:**

This study presents a rule-based RAdiology Data EXtraction tool (RADEX) to enable biomedical researchers and healthcare professionals to automate information extraction from clinical documents. RADEX simplifies the translation of domain expertise into regular-expression models, enabling context-dependent searching without specialist expertise in Natural Language Processing. Its utility was demonstrated in the multi-label classification of fourteen clinical features in a large retrospective dataset (*n* = 16,246) of thyroid ultrasound reports from five hospitals in the United Kingdom (UK). A tuning subset (*n* = 200) was used to iteratively develop the search strategy, and a holdout test subset (*n* = 202) was used to evaluate the performance against reference-standard labels.

**Results:**

The dataset cardinality was 3.06, and the label density was 0.34. Cohen’s Kappa was 0.94 for rater 1 and 0.95 for rater 2. For RADEX, micro-average sensitivity, specificity, and F1-score were 0.97, 0.96, and 0.94, respectively. The processing time was 12.3 milliseconds per report, enabling fast and reliable information extraction.

**Conclusion:**

RADEX is a versatile tool for bespoke research and audit applications, where access to labelled data or computing infrastructure is limited, or explainability and reproducibility are priorities. This offers a time-saving and freely available option to accelerate structured data collection, enabling new insights and improved patient care.

**Key Points:**

***Question*** *Radiology reports contain vital information that is buried in unstructured free-text fields. Can we extract this information effectively for research and audit applications?*

***Findings*** *A rule-based RAdiology Data Extraction tool (RADEX) is described and used to classify fourteen key findings from thyroid ultrasound reports with sensitivity and specificity > 0.95*.

***Clinical relevance*** *RADEX offers clinicians and researchers a time-saving tool to accelerate structured data collection. This practical approach prioritises transparency, repeatability, and usability, enabling new insights into improved patient care*.

**Graphical Abstract:**

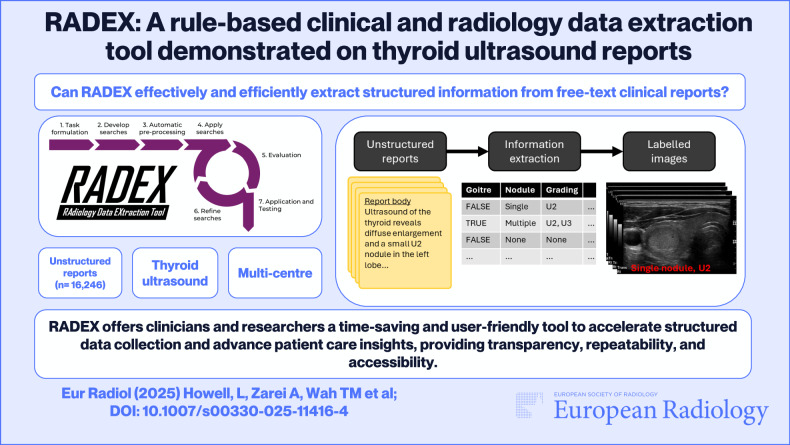

## Introduction

Most information in medical reports is recorded in a narrative ‘free-text’ format [1], allowing reporters to write without restrictions. While this facilitates effective communication [2], it is often necessary to extract and structure relevant data for research and audit purposes [3]. Manual curation (chart review) is time-consuming, tedious, and expensive. Therefore tools that can extract information automatically are valuable for applications such as diagnostic surveillance, case retrieval, cohort building, quality improvement, and research applications [4–6]. In particular, annotating large datasets for training artificial intelligence (AI) models has been identified as a priority for clinical research [7].

Recently, developments in Natural Language Processing (NLP) such as machine learning (ML) and deep learning (DL), especially large language models (LLMs) such as BERT and ChatGPT, have renewed interest in automated information extraction [8, 9]. In particular, domain-specific language models, such as BioBERT [10], ClinicalBERT [11], PubMedBERT [12], and radiology-focused RadBERT [13] have shown promising results for tasks such as text classification, named entity recognition, and summarisation, re-training the base BERT model using clinical text or biomedical literature [14]. For example, in 2022 Wood et al applied BioBERT to classify abnormalities in head MRI reports, achieving an F1-score of 0.962 and 0.930 when evaluated against reference-standard reports and image labels [15]. Also in 2022, Visser et al extracted actionable findings from radiology reports, including X-ray, Ultrasound, CT, and MRI, scoring a sensitivity of 0.841 and specificity of 0.990 [16].

Despite the potential of ML/DL methods, a lack of domain-specific training data and concerns around security, reliability, explainability, and bias have limited their widespread implementation in practice [7, 17]. For these reasons, rule-based methods, which use human knowledge to create computational models, remain popular in medicine [4, 6]. Well-designed rule-based models can achieve similarly high performance to ML/DL techniques [5, 18] while offering improved transparency, applicability, and flexibility. The main challenge of rule-based approaches is that developing robust models is difficult and usually requires interdisciplinary collaboration between technical experts who build the model and clinical experts who provide expertise in the domain of the data [17, 19]. Existing databases such as the Unified Medical Language System (UMLS) [20] or Radiology Lexicon (RadLex) [21] can be applied in ‘dictionary-based methods’ to identify medical terms from large ontologies. However, relying on dictionaries alone often cannot capture the clinical nuances, language variability, and context required for report classification in bespoke research and audit tasks [22]. In these cases, manual knowledge engineering is needed to define custom rules that meet task-specific requirements. These handcrafted rules are usually defined using the established framework of ‘regular expressions’ (regex)—sequences of letters and special characters that define patterns for text-matching [23]. Regex offers an efficient and flexible method for pattern-matching, but expressions are challenging to construct and maintain as there is currently no standardised method for their generation and evaluation [24].

This study presents a novel rule-based tool for RAdiology Data EXtraction (RADEX), which standardises and simplifies the construction of regular-expression models using a high-level syntax and iterative refinement protocol. This enables effective translation of clinical domain knowledge into computational models for automated data annotation and report classification. RADEX encourages a systematic approach to form robust, comprehensive, and reproducible search strategies; iteratively refining regular-expression models to allow reliable data extraction from unstructured radiology reports (X-Ray, MRI, CT, Ultrasound) and other clinical documents (clinical notes, incident reports, pathology, and cytology reports). The tool addresses challenges associated with previous methods, which lack transparent and reproducible reporting, robust evaluation, or rely on access to large labelled datasets [25]. RADEX is aimed at biomedical researchers and healthcare professionals with no specialist prior experience in NLP. It provides a practical, fast, flexible, and free solution that can be integrated with existing clinical systems.

The typical RADEX workflow is demonstrated on a large dataset of thyroid ultrasound reports, a useful example of unstructured reports with variable language [26] and a broad range of diagnostic indications [27]. Thyroid cancer, thyroid nodules, and functional or autoimmune disease have been investigated previously. For example, in 2020 Chen et al used the rule-based tool cTAKES [28] to identify thyroid nodules and classify five characteristics, detecting nodules with 91.9% accuracy on a dataset of 153 reports [29]. In 2022, Dedhia et al also applied cTAKES, classifying 243 ultrasound reports for nine classes with an F1-score of 0.8122 (precision = 0.74, recall = 0.90) [26]. Recently, Pathak et al applied transformer-based models to extract 16 thyroid nodule characteristics in 490 reports, achieving a maximum F1-Score of 0.9495 when considering partial matches on a relatively small dataset of 98 test reports [30]. In this study, 16,246 thyroid ultrasound reports were classified for 14 features, covering common diffuse and focal pathologies as well as nodule gradings. Results were evaluated against 202 reference-standard labels with consensus from three independent expert reviewers. The anonymised dataset produced could be used for purposes such as service evaluation, improving consistency of reporting, or downstream research such as training AI computer vision models [29, 31].

The purpose of this study is to develop and evaluate RADEX, a rule-based data extraction tool that leverages domain expertise to efficiently and accurately extract structured information from free-text clinical reports. The tool aims to provide a scalable, user-friendly, and time-saving solution for information extraction and report classification in large datasets, which is demonstrated on thyroid ultrasound reports.

## Methods and materials

### Dataset

Formal ethical approval was waived for this retrospective study of collated clinical and imaging reports in accordance with the Institutional Health Research Authority Framework. All radiology reports used in this study were obtained from a retrospective dataset, including all examinations tagged Ultrasound Neck (UNECK), Ultrasound thyroid (UTHYD), or Ultrasound thyroid and parathyroid (UTHPY), extracted from the Computerised Radiology Information System (CRIS) between 01/01/2015 - 31/12/2019 across five different hospitals in the same geographical area in the United Kingdom. Paediatric patients ($<$ 18 years) were excluded, and multipart examinations were merged into a single pseudoidentifier. Where multiple reports were present for the same pseudoidentifier, only the most recent report was taken to avoid any possibility of data leakage. This resulted in a dataset comprising 16,246 reports from unique patients. All reports were in an unstructured free-text format and included clinical history, report body, and findings, with embedded headers dividing these sections. The average (mean) total report length was 93 words, and the dataset contained reports written by 222 unique reporters with varying roles and experience, including general or specialist sonographers, radiology trainees, and general or specialist certified radiologists.

To develop the search strategy, a subset of 200 reports was sampled at random as a tuning set. A further 202 reports were sampled as a holdout test dataset, allowing unbiased evaluation of the expected performance and ensuring there was no overfitting. These 402 reports were annotated independently by three reviewers: an experienced radiologist (UK consultant grade; US attending equivalent) and two foundation doctors with experience reading and interpreting radiology reports. In ambiguous cases, the experienced radiologist served as the adjudicator for reference-standard labels, reviewing the report’s associated ultrasound images using the Patient Archive and Communication System (PACS) as necessary to ensure the most accurate annotations possible. Fourteen classes were chosen to cover the features of interest, including common diffuse and focal pathologies observed in ultrasound examination of the thyroid [32–34]. Since reports were from the UK, British Thyroid Association (BTA) gradings were used to stratify thyroid nodules based on their malignancy risk [35].

The classes chosen for classification were:

- Thyroid examination—thyroid examination conducted.
- Lymph node examination—lymph node examination conducted.
- Thyroid nodule(s)—one or more thyroid nodules.
- Multiple thyroid nodules—multiple discrete nodules or multinodular goitre (MNG).
- Solitary thyroid nodule—finding of thyroid nodule(s) but not multiple nodules or MNG.
- Altered thyroid echotexture—indicative of diffuse thyroid disease such as thyroiditis.
- Goitre - enlarged thyroid, including simple and multinodular goitre.
- Previous thyroid surgery—including hemi and total thyroidectomy.
- BTA U1—British Thyroid Association grading for normal thyroid
- BTA U2—British Thyroid Association grading for benign thyroid nodule
- BTA U3—British Thyroid Association grading for indeterminate/equivocal thyroid nodule
- BTA U4—British Thyroid Association grading for suspicious thyroid nodule
- BTA U5—British Thyroid Association grading for malignant thyroid nodule
- Normal thyroid - mention of thyroid examination but no finding of thyroid nodule(s), altered echotexture, goitre, or previous thyroid surgery.

### RADEX tool

The RAdiology Data EXtraction (RADEX) tool is a general-purpose software package aimed at medical professionals and biomedical researchers who require a simple method to extract high-quality structured datasets from free-text clinical reports. RADEX enables rule-based extraction of relevant information, constructing regular-expression models to classify reports and provide interpretable predictions.

Regular expressions are a standard technique implemented in many programming languages (e.g. Python, Perl, Java, Ruby), which offer flexible and efficient searching in large datasets [24]. These expressions are sequences of characters and special symbols that explicitly define searches, including wildcards, character classes, quantifiers, and boundary matches, which are useful when searching for medical terms in context [23]. For example, in Python the regular expression (1) ‘\bthyr\w*\b’, the ‘\b’ represents a word boundary, ‘\w’ matches one or more word characters (letters or numbers), and ‘*’ means ‘zero or more times’. This pattern would match any word that starts with ‘thyroid’, such as ‘thyroid’, ‘thyroidectomy’, or ‘thyroiditis’. In the expression (2) ‘\bthyroid\b(?:\W + \w + ){0,3}?\W + \bnodules\b | \bmultinodular\b’, the non-capturing group ‘(?:\W + \w + ){0,2}’ matches 0 to 2 words between ‘thyroid’ and ‘nodules’, and the ‘|’ means ‘OR’, meaning the expression matches the word ‘multinodular’ as well as phrases such as ‘thyroid nodules’ and ‘thyroid contains multiple nodules’. While this framework allows highly flexible pattern-matching, regex is complex to learn and becomes difficult to interpret as the complexity of patterns increases.

**Fig. 1:**
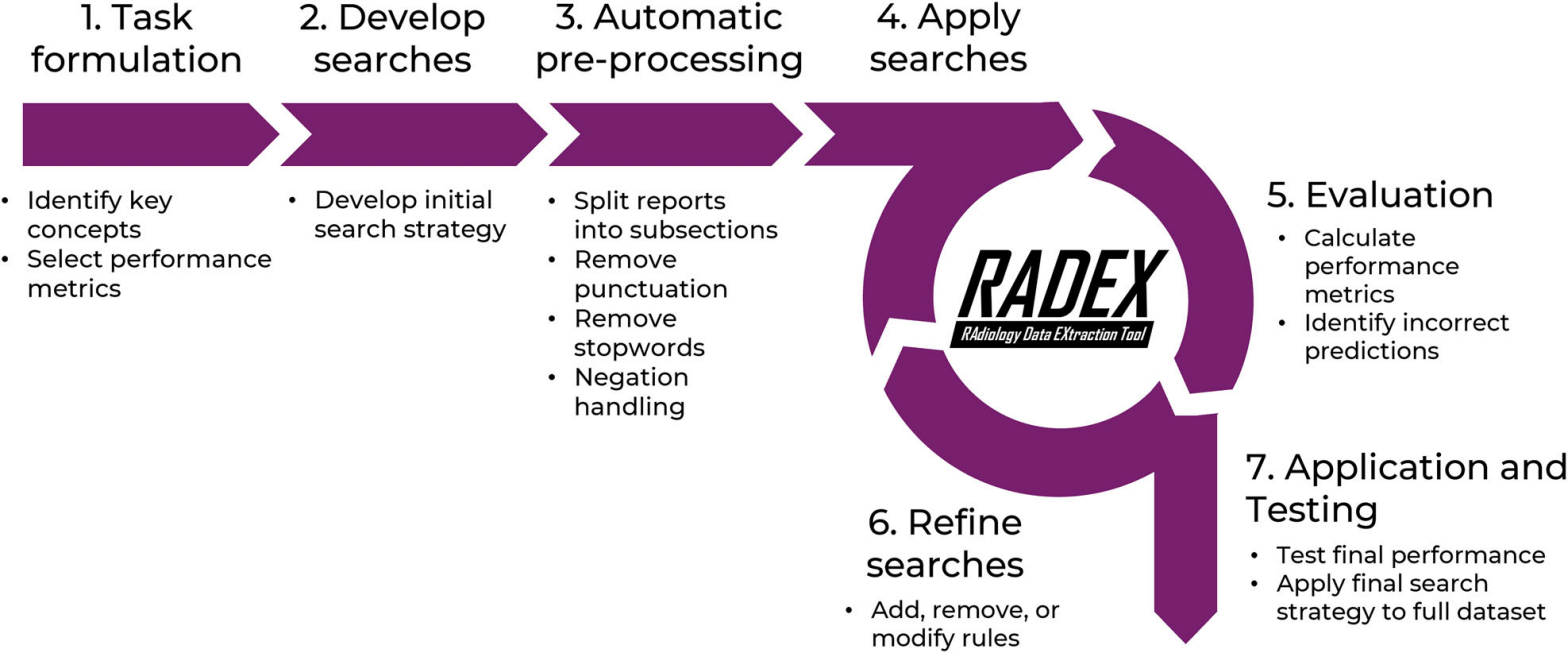
Typical workflow for the Radiology Data Extraction Tool (RADEX), including iterative development of searches

RADEX centres around a user-friendly ‘search strategy’, which is created with a high-level syntax of keywords, modifiers, and logical connectors. For example, in RADEX, the expressions ‘thyr*’ and ‘thyroid NEAR/2 nodules OR multinodular’ are equivalent to regular expressions (1) and (2) above. This enables non-expert users to create robust regular-expression-based models that consider context, differentiate between similar terms, and account for variations in spelling, phrasing, and report structure. This approach to developing a search strategy takes inspiration from popular search tools for biomedical literature databases such as PubMed Advanced Search Builder and Ovid Advanced Search, which are familiar to many biomedical researchers and medical professionals, lowering the barrier to entry for using the tool. Similarly to the literature search process of systematic reviews, the RADEX methodology promotes rigour, comprehensivity, and reproducibility in data extraction [36]. A diagram of the typical RADEX workflow is shown in Fig. 1, and each step is described in more detail in the sections below.

RADEX was developed in Python 3.9 and is available for Windows, MAC, and Linux OS. The source code for the project is available on GitHub under the BDS 3-Clause Licence, allowing its free use and modification. Documentation, installation instructions, and instructions for developers are available online. Also included is a labelled dataset of 110 synthetic thyroid ultrasound reports, generated using OpenAI’s ChatGPT-3.5 [37]. This provides a relevant quick-start example of the RADEX workflow on data that can be readily shared, including a demonstration of a prototype graphical user interface (GUI).

#### Task formulation

The first stage for information extraction is task formulation, where the key requirements and datasets are identified. This includes defining the clinical question, population of interest, and concepts needed to classify reports. For best performance, the task requirements should be sufficiently narrow and well-defined to ensure precise mapping between the tool’s outputs and the intended clinical concepts, reducing ambiguity and improving model performance. The user should also consider which classification metrics are most relevant for performance evaluation, depending on the task requirements and characteristics of the dataset [38, 39].

#### Develop search strategy

The search strategy is then defined using RADEX’s simple but flexible syntax, which leverages domain knowledge to extract structured information without requiring the user to have prior expertise in NLP or regular expressions. This RADEX search strategy comprises keywords, modifiers, and logical connectors which define clinical concepts.

Keywords include synonyms, abbreviations, and other terms related to the concept, such as medications, procedures, and diseases. Relevant keywords can be identified using expertise in the domain of the data or using existing biomedical ontologies such as UMLS [20] and RadLex [21]. Modifiers broaden the search scope, accommodating variations in spelling, pleuralised words, and typographical errors. For example, the asterisk (‘*’) modifier represents any number of characters, which is useful since many related clinical terms share a common root, suffix, or prefix, originating from their Greek or Latin etymology [23]. For example, ‘neur*’ will match a variety of terms relating to nerves and the nervous system, such as ‘neuron’, ‘neuropathy’, and ‘neurofibromatosis’. Similarly, ‘*therap* would match ‘radiotherapy’ and ‘therapeutic’. The question mark (‘?’) represents any optional single character, most useful for accommodating pluralisation and spelling variation. For example, ‘h?emoglobin’ would match ‘haemoglobin’ and ‘haemoglobin’, and ‘wom?n’ would match ‘woman’ or ‘women’. The order-independent and order-dependent proximity operators ‘NEAR/X’ and ‘THEN/X’ identify terms that are separated by ‘X’ or fewer words. For example, ‘thyroid NEAR/2 *nodul*’ matches phrases such as ‘thyroid nodules’, ‘multinodular thyroid’, or ‘thyroid contains multiple nodules’. Expressions can be combined using the logical connectors ‘AND’, ‘OR’, ‘NOT’ and ‘EXCEPT’ (equivalent to ‘AND NOT’) to build complete definitions for concepts. For example, a expression for identifying thyroid nodules could be: ‘thyroid NEAR/2 *nodul* OR (node? EXCEPT lymph node?)’. Here a search for the word thyroid in proximity to nodules is combined with a search for node/nodes, excluding lymph nodes. Combining these expressions allows the development of search strategies to reliably classify increasingly complex concepts.

#### Pre-processing

Before running the search strategy, the reports are pre-processed to ensure consistent results. Filtering can be applied to enforce inclusion/exclusion criteria such as date ranges, or patient demographics such as age. Reports are then split into relevant sections based on selected headers, and the text is then standardised: removing stopwords, selecting punctuation, trailing whitespace, and converting the text to lowercase. Sentence tokenisation is applied to break the text into individual sentences. Negated phrases are handled using Chapman’s NegEx algorithm [40]. This uses a clinically relevant list of pre-determined modifiers to identify negation modifiers and their scope. For example, the phrases ‘there are no thyroid nodules’ and ‘thyroid nodules were not observed’ are negated by the pre- and post- negation terms ‘no’ and ‘not’ and are automatically excluded from potential matches.

#### Apply search strategy

To process the text, the RADEX search strategy is converted to a regular-expression model in the software backend. A grammar function represents the permitted operators and their precedence (NOT $>$ AND $>$ OR), detecting unmatched parentheses, unexpected symbols, or incorrect syntax. Once verified, the search strategy is recursively evaluated, converting each sub-expression to a regular-expression applied to search the text. Matching words and phrases are extracted, and their positional indexes can be used to highlight the report. Depending on the presence or absence of matches and the applied logic, reports are classified for one or more labels. This process, similar to an inference engine, enables traceability at the report level, enhancing explainability and facilitating the iterative development of searches.

#### Evaluation

The model is then evaluated using an annotated tuning subset of the data. Label-based metrics such as classification accuracy, sensitivity (true positive rate, recall), specificity (true negative rate), precision (positive predictive value), and F1-score (harmonic mean of precision and recall) are calculated to assess the quality of the search strategy. In cases where multiple labels are assigned to each report, the macro- and micro-averaged metrics, in addition to the example-based Hamming loss (total fraction of wrong labels) and exact match ratio (percentage of reports with all labels predicted correctly) are calculated [41, 42]. In all cases, no single metric captures all important information, and the most useful metrics depend on the study objectives and dataset characteristics [38].

#### Refine search strategy, application, and testing

The search strategy is then refined based on a review of incorrect predictions in the tuning set, adding, removing, or modifying rules as necessary to build a complete search strategy. RADEX identifies false positive and false negative predictions using reference-standard labels, highlighting matching phrases within the report body to assist this refinement process. The improved searches are then re-applied and re-evaluated on the tuning set, improving the search strategy over several cycles. Once satisfactory performance is achieved, the searches should be assessed on a holdout test dataset. This allows unbiased quantification of the expected performance on the full dataset, assessing the generalisability of the model and ensuring there is no overfitting.

### RADEX for thyroid ultrasound reports

While RADEX is a general tool for clinical information extraction, this study demonstrates its application in the context of thyroid ultrasound reports. Combining multi-label classification ultrasound reports with linked ultrasound images enables the curation of a large labelled dataset of thyroid ultrasound scans, suitable for developing computer vision models in the future. The primary metric for assessing RADEX performance on this dataset was the F1-Score, which was chosen since both high precision and recall were important. A target micro-average F1-score of 0.90 was selected based on the study requirements and the inter-annotator agreement.

Twelve classes were searched directly in the report body, including thyroid examination, lymph node examination, thyroid nodule(s), multiple thyroid nodules, altered thyroid echotexture, goitre, and BTA U1-U5. In addition to the report body, the clinical history was included in searches for previous thyroid surgery. The classes solitary thyroid nodule and normal thyroid were derived from using simple logic, where mention of thyroid nodule(s) and not multiple thyroid nodules implies a single nodule, and normal thyroid is defined as the absence of any other focal or diffuse thyroid abnormality. Simple post-processing rules were applied to ensure consistency in the final dataset, for example, finding a BTA U2 to U5 grading must imply the presence of a thyroid nodule. Once satisfactory scores for the sensitivity and specificity were achieved, the same searches were applied to the test dataset to ensure that the terms used were general enough to work on new data. The final search strategy is shown in Table 1.

**Table 1:** Search strategy for neck and thyroid ultrasound dataset

Class	Search strategy
Thyroid examination	thyroid*
Lymph node examination	lymph* | (node & ¬thyroid* node),
Thyroid nodules(s)	(nodul* & ¬(nodul*∼2cartilage | nodul*∼2parotid | nodule*∼2salivary | nodul*∼2submandibular)) | (cyst* & ¬(thyroglossal cyst | cyst*~2parotid | cyst*~2salivary | cyst*~2submandibular | cyst*~1sebaceous | cyst*~1inclusion | cyst*~1lymph* | cyst*~1node)) | thyroid~4lesion* | thyroid~1node* | adenoma
Multiple thyroid nodules	(nodules & ¬(nodules∼2cartilage | nodules∼2parotid | nodules∼2salivary | nodules∼2submandibular)) | (cysts & ¬(thyroglossal cysts | cysts~2parotid | cysts~2salivary | cysts~2submandibular | cysts~2sebaceous | cysts~2inclusion | cysts~2lymph* | cysts~2node)) | thyroid~4lesions | thyroid~1nodes | multi?nodul* | nodul*~2both | other~2nodul* | second*~2nodul* | (superior~3nodul* & inferior~3nodul*) | (nodule*~3left & nodule*~3right) | (nodule*~3left & nodule*~3isthmus) | (nodule*~3right & nodule*~3isthmus) | mng
Altered thyroid echotexture	thyroiditis | grav?s | heterogen*~2echo* | heterogen*~2thyroid | heterogen*~1texture | inflamed*
Goitre	goit?r? | mng | multi?nodul* thyroid | enlarged~2thyroid | (enlarged~2gland & ¬enlarged~2parathy*) | enlarged~2lobe | hyperplasia
Previous thyroid surgery	thyroidectomy | lobectomy | isthmusectomy | (surgery & ¬refer*~2surgery) | resect* | incomplete~2thyroid | post?op | bed | hemiagenesis
BTA U1	u1
BTA U2	u2 & ¬(u2?u3 | u2 ~ 1 ?3)
BTA U3	u3 & ¬(u3?u4 | u3 ~ 1 ?4 | u2?u3 | u2 ~ 1 ?3)
BTA U4	u4 & ¬(u4?u5 | u4 ~ 1 ?5 | u3?u4 | u3 ~ 1 ?4)
BTA U5	u5 & ¬(u4?u5 | u4 ~ 1 ?5)
Solitary thyroid nodule	“Thyroid nodules(s)” & ¬”Multiple Thyroid Nodules”
Normal thyroid	“Thyroid examination” & ¬(“Thyroid nodule(s)” | “Altered thyroid echotexture” | “Goitre” | “Previous thyroid surgery”)

The symbols &, |, ¬, and ∼X, are shorthand for ‘AND’, ‘OR’, ‘NOT’, and the bidirectional word proximity operator NEAR/X, with adjacency X as an integer. The modifiers ‘*’ and ‘?’ are wildcard modifiers

The initial search strategy was constructed with interdisciplinary input from experienced head and neck radiologists as well as UMLS. These searches were then applied to the clinical dataset, and entries where there was disagreement between the reference standard and predicted labels were flagged for manual review. The search criteria were iteratively refined in several cycles, adding or removing phrases from the search strategy to reduce the number of incorrect predictions. All computation was conducted on a research laptop with average specifications (AMD 4600H CPU, 16GB RAM).

## Results

In the reference-standard labels, the average number of labels per report (cardinality) was 3.06, and the percentage of possible labels assigned on average per report (label density), was 34%. The inter-annotator reliability was assessed using Cohen’s Kappa, which was 0.94 for rater 1 and 0.95 for rater 2, indicating a strong agreement with the adjudicator. The time taken to annotate 200 reports was approximately 2.5 h per annotator, which if extrapolated to the full dataset, suggests approximately 203 h of manual labelling would be needed per annotator.

**Fig. 2:**
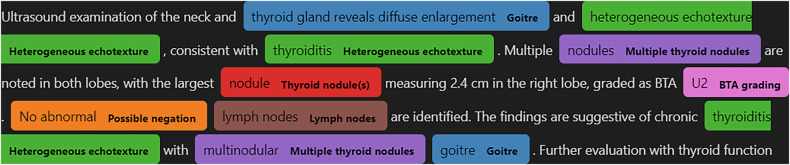
A synthetic neck and thyroid ultrasound report, highlighted using a rule-based radiology data extraction tool. This example was correctly classified as positive for goitre (blue), altered (heterogeneous) thyroid echotexture (green), multiple thyroid nodules (purple), and lymph node examination (brown). The phrase ‘no abnormal’ was matched as a possible negation modifier to the term ‘lymph nodes’

**Fig. 3:**
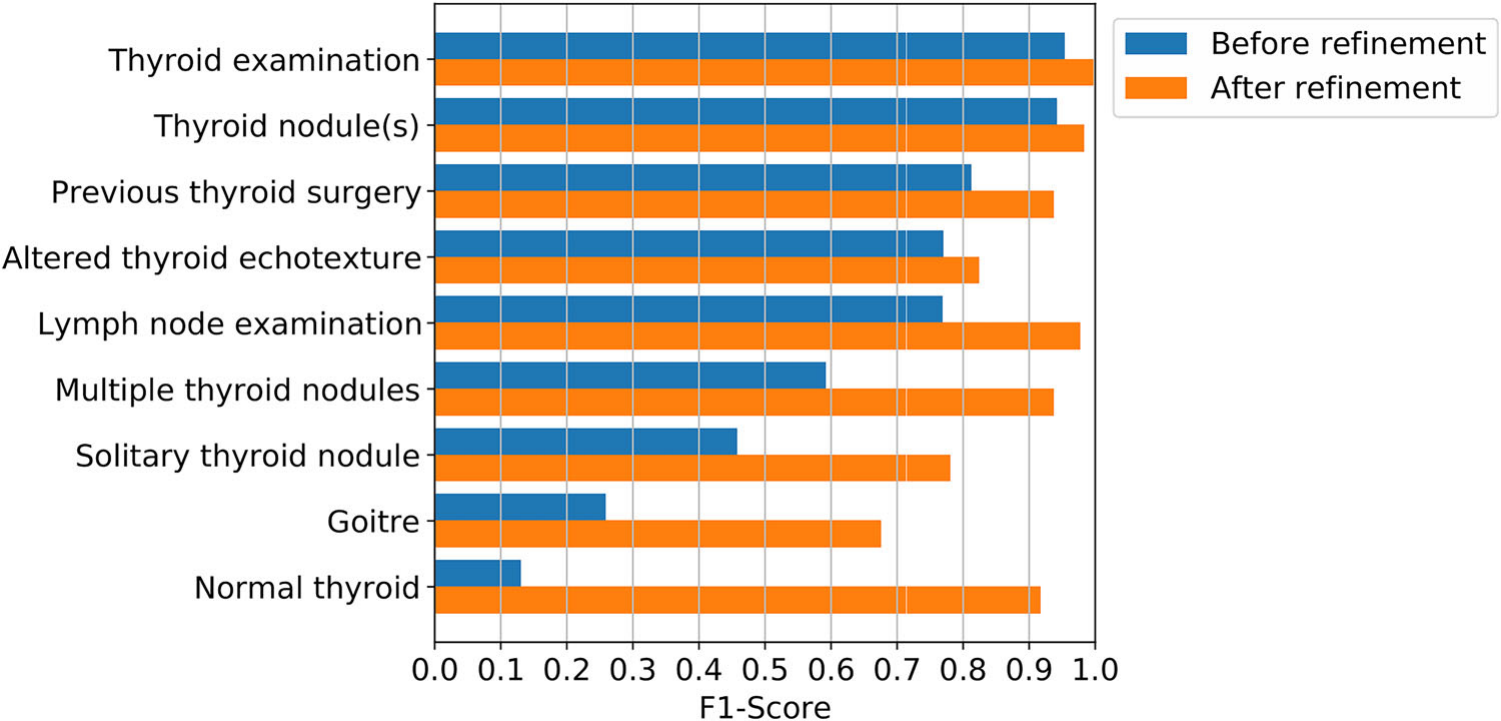
F1-score for rule-based predictions on the holdout test clinical dataset, before and after refining the searches

Applying the RADEX search strategy in Table 1 enabled classification and highlighting of the reports for the 14 different classes (Fig. 2). Applying the iterative tuning approach to the development of the search strategy increased the F1-score across all classes (Fig. 3), improving the micro-average by 0.222. Generally, false positive predictions arose from semantic context errors, negated, and hypothetical sentences, while false negatives were due to missing keywords and negation errors.

Using RADEX, the Hamming loss for the nine main classes (excluding the BTA gradings) was 0.0418, suggesting, on average, less than 5% of the total labels were incorrectly predicted. The harshest metric, the exact match ratio, was 0.733, showing the percentage of reports with all nine classes correct. Label-based metrics and their micro- and macro-averages are given in Table 2, scoring each class individually, with all classes except solitary thyroid nodule and goitre having F1 scores < 0.8. Semantically, solitary thyroid nodule was the most complex concept to predict, since it was difficult to distinguish cases where a single nodule was mentioned multiple times, from cases with multiple nodules present in the thyroid. Notably, goitre had a high sensitivity with no false positives in the test set but lower precision. This is because of the conservative approach taken to labelling this class and the imprecise language used in its reporting. For example, phrases such as ‘thyroid gland appears slightly enlarged’ were commonly predicted as false positives, requiring additional context outside the report body to predict these cases accurately. Overall, the micro-average sensitivity and specificity across the nine classes were 0.971 and 0.957, respectively, exceeding the targets set at the start of the study.

**Table 2:** Scores for multi-label classification of neck and thyroid ultrasound reports with regular expressions

	Precision (PPV)	Sensitivity (Recall/TPR)	Specificity (TNR)	F1-score
Thyroid examination	0.994	1.000	0.978	0.997
Normal thyroid	0.846	1.000	0.990	0.917
Previous thyroid surgery	0.902	0.974	0.976	0.937
Thyroid nodule(s)	0.966	1.000	0.954	0.983
Multiple thyroid nodules	0.943	0.932	0.956	0.937
Solitary thyroid nodule	0.719	0.852	0.949	0.780
Altered thyroid echotexture	0.824	0.824	0.984	0.824
Goitre	0.509	1.000	0.851	0.675
Lymph node examination	0.982	0.971	0.900	0.977
Micro-average	0.917	0.971	0.957	0.943
Macro average	0.854	0.950	0.949	0.892

**Fig. 4:**
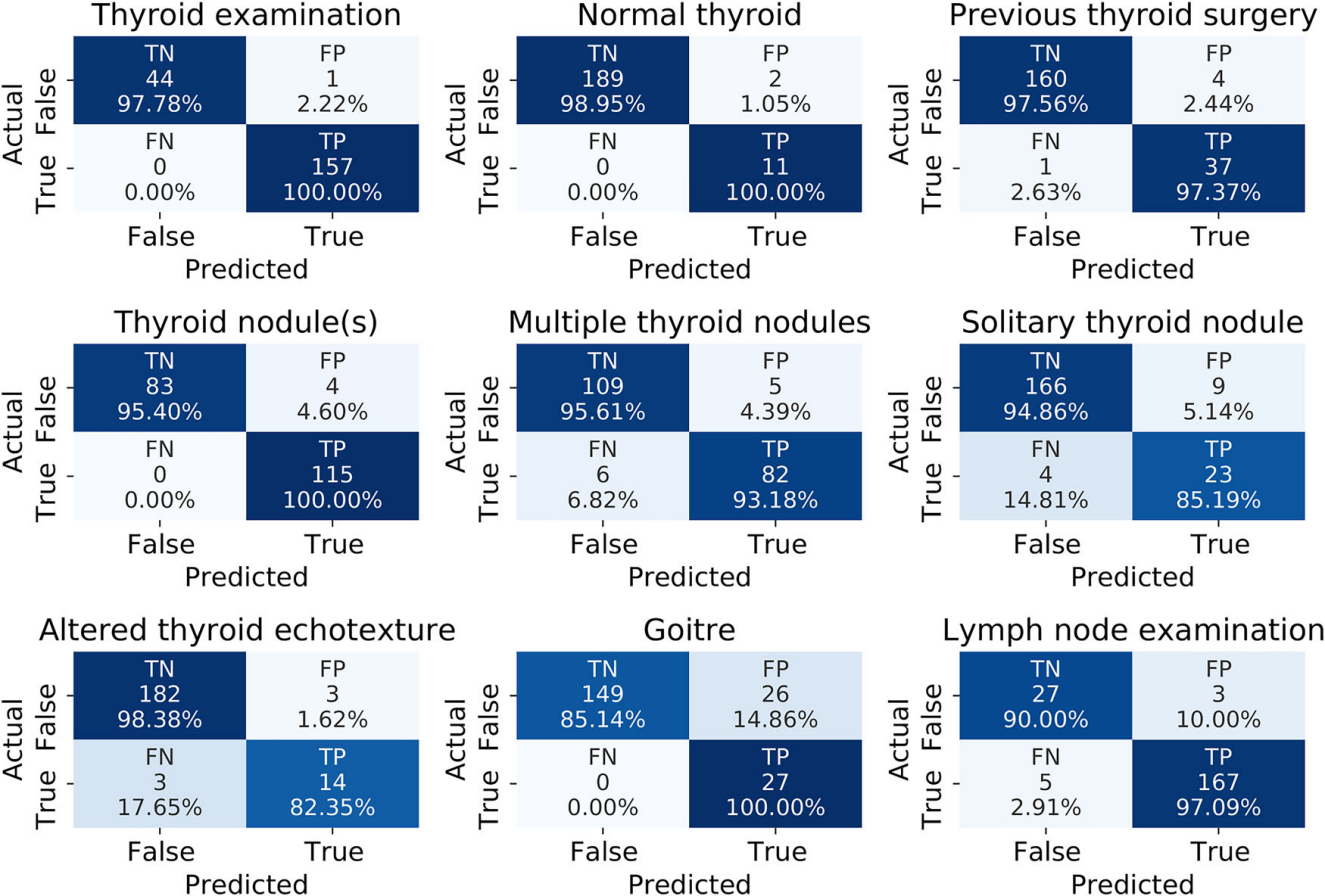
Class-wise confusion matrices for multi-label data. True positive (TP), True negative (TN), False positive (FP), and False Negative (FN) counts and normalised scores for each class

Importantly, the recall was generally high, meaning the rate of false negatives was low. This is significant since a manual review of positive predictions in the predictions can eliminate false positives, but finding missed reports among the full dataset (false negatives) requires a review of more total reports. The true and false positives and negatives are visualised with confusion matrices in Fig. 4. In addition to these nine classes, the BTA gradings for thyroid nodules were predicted well, with micro-average F1-Score of 0.967.

This method is also relatively computationally inexpensive, requiring no specialist hardware or cloud infrastructure. The total processing time for 16,246 reports was 200s, or 12.3 ms per report. As expected, processing time increased linearly with the number and average length of the reports, as well as increasing with the number and complexity of searches.

## Discussion

This study presented RADEX, a rule-based data extraction tool for classification and information extraction in clinical free-text reports. This was demonstrated on a large dataset of 16,246 thyroid ultrasound reports, classifying 14 clinical findings and scoring a micro-average sensitivity of 0.971 and specificity of 0.957, which exceeded the targets set for the study. RADEX processed the full dataset in just 200 seconds, which represented a large time-saving benefit when compared to the 203 h estimated for manual annotation. Thyroid ultrasound reports presented an interesting case study for classification due to their variable language [26] and a broad range of clinical concepts [27]. Compared with similar work applying NLP to thyroid ultrasound reports, the methods used improved on previous rule-based approaches and performed approximately as well as DL methods. Using dictionary-based approaches to classify thyroid nodules in reports, Dedhia et al reported an F1-score of 0.812 [26], and Chen et al scored 0.919 in accuracy [29]. This study achieved an F1-Score of 0.943 and an accuracy of 0.980. The F1-Score was also within one percentile of the DL transformer-based approach reported by *Pathak* et al which was 0.945 [30]. However, it should be noted that since these studies use different datasets and class definitions, it is difficult to compare these results directly. In this study, the micro-average F1-score of 0.943 illustrates a low false positive and false negative rate, which is important since previous approaches using handcrafted regular expressions often perform poorly in recall [24]. This improvement was attributed to the flexible, iterative refinement process used, enabling the creation of custom rules that were sufficiently general to capture language variations among the 222 reporters and five different healthcare organisations in the dataset.

The main advantages of RADEX are that accurate results can be obtained quickly, results are transparent and reproducible, and the tool can be used for free with no special hardware requirements. The tool prioritises usability, leveraging the power of regular expressions but without requiring the user to understand their complex syntax. This simplifies the process of creating robust regular-expression models, allowing users to annotate large datasets with no specialist NLP expertise. The iterative refinement process ensures the search strategy is comprehensive, and the independent evaluation ensures the final model is generalisable within the domain of the dataset. The final search strategy can also be easily exported and shared without compromising patient confidentiality, supporting reproducibility, which is critical in clinical NLP applications [25].

Compared with existing methods, RADEX provides greater flexibility than dictionary-based approaches and better explainability than DL methods at the cost of requiring the search strategy to be manually defined. Dictionary-based methods can extract recognised concepts such as disease names, symptoms, and treatments from clinical ontologies. However, they struggle with capturing specific, context-dependent, or nuanced information, as dictionaries alone often cannot capture all required patterns to define a concept [22]. ML/DL methods automatically learn patterns and relationships by analysing text datasets, allowing for more accurate extraction of nuanced information. However, they require large labelled datasets, expensive computational resources, and often lack transparency, making it difficult to explain or reproduce their outputs in clinical settings. RADEX bridges these gaps by simplifying the process of defining and refining custom rulesets. This makes it suitable for standalone use or in conjunction with existing dictionary-based or ML/DL methods. For example, RADEX could be used to supplement dictionary-based methods in cases of difficult-to-extract concepts, or to provide an explainable baseline for DL/ML models to be evaluated against.

This explainability is important to support decision-making [6] and to satisfy ethical considerations and data governance standards related to accountability [43]. While regular-expression models alone can be difficult to decipher; the high-level syntax used to develop the RADEX search strategy is easy to understand, aiding interpretability of model predictions. This is a major advantage compared with ML/DL methods, where the non-linear operations in ‘black box’ DL models make explainability difficult [44]. In particular, since LLMs rely on statistical patterns in their training data, they lack a true understanding of underlying medical concepts, which can lead to inaccurate predictions [45]. The human-created search strategy of RADEX can be modified to correct specific errors, meet changing requirements, or adapt to terminology changes in different regions or over time [2]. This is not the case with DL models, which require re-training using additional labelled training. This is resource-intensive and does not guarantee performance improvement. Importantly, RADEX is also scalable to large datasets and has no special hardware requirements. This helps to alleviate concerns regarding cost and data security since all processing is performed locally, rather than relying on secure cloud computing infrastructure, which restricts the use of AI in clinical settings [5].

The main limitation of the RADEX method is its limited applicability to complex semantic tasks, where understanding the wider context and the subtlety of human language is important. In these cases, DL or hybrid techniques may be more appropriate [19], but rule-based approaches are still useful as a baseline to justify the associated implementation challenges and explainability tradeoff [6]. Inter-annotator agreement also presents a challenge, where different interpretations of the same data can introduce uncertainty [23]. This is reflected by the inter-annotator agreement (Cohen’s Kappa = 0.94), showing a minority of reports containing ambiguous findings that required cross-referencing with clinical images in the reference-standard dataset. While this approach minimises uncertainty, it does limit the theoretical maximum prediction accuracy as this information is not available to the model, requiring a multimodal approach. A better strategy may have been to quantify uncertainty using a method such as NegBio [46], allowing a secondary review of unclear cases. Furthermore, while test set metrics give an unbiased estimate of the expected performance on the larger dataset, this may not represent the wider generalisability to external datasets, since language use can vary significantly between institutions and over time. Since data was collected in the UK, all reports were in British English, and thyroid nodule malignancy risk was given by the British Thyroid Association (BTA) grading system. However, similar principles could be applied to reports in other languages or different grading systems, such as the European Thyroid Association (ETA) and American College of Radiologists (ACR) Thyroid Imaging Reporting and Data System (TIRADS) gradings [47, 48]. This would require further refinement of searches, which can be achieved using the iterative RADEX method.

Overall, RADEX provides a practical alternative to dictionary-based and ML/DL methods for free-text information extraction, effectively leveraging clinical expertise to create custom rule-based models for bespoke research and audit tasks. This was demonstrated in the classification of thyroid ultrasound reports but could be similarly applied to other radiology reports and clinical documents such as clinical notes, incident reports, pathology, and cytology reports in the future. Micro-average sensitivity, specificity, and F1-Score were 0.971, 0.957, and 0.943, respectively, and the Hamming loss was 0.0418. No specialist hardware was required, and the technique was shown to be scalable to large datasets, taking 12.3 ms/report. This method offers a practical alternative or precursor to other approaches, such as ML and DL, especially where access to labelled training data, computing infrastructure, explainability, or reproducibility are concerns. This provides a time-saving and freely available tool for future studies in clinical decision support, quality improvements, unbiased identification of disease cohorts for research studies, query-based case retrieval, and research tasks such as annotating medical images for AI analysis.

## Abbreviations

AI: Artificial intelligence
DL: Deep learning
ML: Machine learning
MNG: Multinodular goitre
NLP: Natural Language Processing
RADEX: Radiology data extraction tool
Regex: Regular expressions
UMLS: Unified medical language system
